# Fresh Platelet-Rich Plasma Gel Reduces Sciatic Nerve-Associated Neuropathic Pain in Rats by Suppressing Tumor Necrosis Factor-α and Interleukin-6 Expression

**DOI:** 10.7759/cureus.90019

**Published:** 2025-08-13

**Authors:** Michiaki Mukai, Kentaro Uchida, Masayuki Miyagi, Gen Inoue, Hiroyuki Sekiguchi, Yuji Yokozeki, Yasuhiro Shiga, Kazuhide Inage, Eguchi Yawara, Sumihisa Orita, Masashi Takaso, Seiji Ohtori

**Affiliations:** 1 Department of Orthopedic Surgery, Chiba University, Graduate School of Medicine, Chiba, JPN; 2 Department of Orthopedic Surgery, Kitasato University, School of Medicine, Sagamihara, JPN; 3 Medical Sciences Research Institute, Shonan University, Chigasaki, JPN; 4 Center for Frontier Medical Engineering, Chiba University, Chiba, JPN

**Keywords:** allodynia, neuropathic pain, peripheral nerve injuries, platelet products, platelet-rich plasma

## Abstract

Objective: Nerve wrapping using biomaterials has shown promising results in promoting nerve regeneration and improving functional outcomes in revision surgery cases. Our previous study on the local administration of basic fibroblast growth factor via a carrier demonstrated a limited effect. Thus, we have focused on platelet-rich plasma (PRP), a treatment material with recent evidence of tissue repair effects, and on a method of wrapping injured nerves by gelatinizing PRP without a carrier. However, there are few reports on studies targeting peripheral neuropathic pain. This study aimed to examine the efficacy and mechanism of action of this method.

Materials and methods: Wistar rats were employed as a model of chronic constriction injury (CCI) and divided into two groups: CCI (untreated) and PRP-G (treated with PRP gel wrapping). PRP gels were prepared by centrifuging peripheral blood from other rats, followed by activation. Pain behavior test and real-time quantitative polymerase chain reaction of sciatic nerve RNA were conducted on postoperative days (PODs) three, seven, and 14 to quantify tumor necrosis factor-α (TNF-α) and interleukin-6 (IL-6) (*Tnf-α and Il-6*) gene expression.

Results: Pathological evaluations of sciatic nerves performed on POD seven to investigate the neuroprotective effects of PRP gel wrapping revealed a significantly higher density of myelinated axons in the PRP-G group. In the CCI group after POD three, the pain threshold decreased to 0.88 ± 0.22 g, which was approximately one-third of the pre-treatment level, while the PRP-G group showed a pain threshold approximately twice that of the CCI group. Quantitative PCR results showed that, compared to untreated rats, both groups exhibited significantly increased expression of *Tnf-α* and *Il-6* in the sciatic nerve after POD three. However, at POD seven and POD 14, the increase in expression was significantly reduced to approximately half that of the CCI group in the PRP-G group.

Conclusions: PRP gel wrapping has a substantial long-term suppression effect on neuropathic pain, possibly involving suppression of *Tnf-α* and *Il-6* expression in the nerves. This study suggests that PRP gel wrapping may be clinically applicable as an adjunctive therapy for peripheral nerve injury.

## Introduction

Peripheral nerve entrapment, also known as compression neuropathy, can manifest as paresis, paresthesia, and pain. Nerve entrapment syndrome is rarely life-threatening, but it can significantly impair a patient’s quality of life and ability to function. The most common chronic nerve compression injuries include carpal tunnel syndrome, cubital tunnel syndrome, and tarsal tunnel syndrome [1-4]. If the condition is refractory to conservative therapy, nerve decompression surgery is performed. After surgery, fibrosis and adhesion to surrounding scar tissue can be reduced by protecting and isolating nerves from the surrounding tissues [5].

Recurrence of symptoms can sometimes occur following initial surgery, requiring revision procedures. Nerve wrapping with biomaterials or artificial materials after dissection or nerve repair procedures is a common technique utilized in recurrent or refractory situations and has been extensively investigated [6]. Animal models of sciatic nerve loss or nerve crush injury have been used to demonstrate electrophysiological or pathological nerve recovery by wrapping [7-9].

Peripheral nerve therapeutic materials using single growth factors and effective drugs have been studied, mainly for nerve regeneration, with satisfactory results [10-12]. Additionally, in our previous study, we have shown earlier that in a rat model of chronic constriction injury (CCI), collagen sheets supplemented with basic fibroblast growth factor (bFGF) can ameliorate neuropathic pain [13]. However, this effect was limited; therefore, we considered using platelet-rich plasma (PRP), which contains numerous growth factors, to wrap the damaged nerves.

PRP is obtained by centrifuging peripheral blood to concentrate platelets and has been shown to have tissue regeneration and anti-inflammatory properties because of the numerous growth factors and cytokines present in the platelet alpha-granules [14]. The application of PRP is being explored in medical fields beyond orthopedics [15-17]. Moreover, both basic and clinical studies are actively underway across a wide range of orthopedic conditions, including peripheral neuropathies [18-20]. PRP is predicted to offer mid-term efficacy, because there are sporadic reports of long-lasting effects in patients with mild to moderate carpal tunnel syndrome, a common peripheral neuropathy, lasting several months or longer following a single local injection [21-23].

Activated PRP can form a gel [24,25], which can be wrapped around nerves for local delivery and is naturally absorbed, making it a promising treatment for peripheral neuropathy. Several animal model studies have demonstrated that PRP promotes the regeneration of injured peripheral nerves. Most studies have used PRP-filled conduits for neural deficit models [26,27]. In contrast, few animal experimental studies have reported the use of PRP or PRP gel administration in neuropathic pain-induced models.

In this study, we investigated the mechanism of action of PRP gel on peripheral nerve-derived pain, focusing on tumor necrosis factor-α (TNF-α) and interleukin-6 (IL-6), which have recently attracted attention in acute and chronic neuropathic pain [28]. In our previous study, we also reported that the expression of TNF-α and IL-6 in the sciatic nerve decreased after nerve release and dissection in a rat sciatic nerve constriction injury model [29].

Here, using a rat CCI model, we investigated the effects of PRP gel wrapping on neuropathic pain and its possible mechanisms.

## Materials and methods

Experimental animals

Male Wistar rats weighing 240-400 g were used for the study (CLEA, Tokyo, Japan). All protocols for animal experiments (reference number: 4-268) were approved by our university’s ethical boards and carried out in accordance with the National Institutes of Health’s Guidelines for the Care and Use of Laboratory Animals (2011 Revision). Additionally, the ARRIVE (Animal Research: Reporting of In Vivo Experiments) guidelines have been used to report this work.

A semi-barrier housing system with a 12-hour light/dark cycle, 21-23°C, 45-65% humidity, and a standard rodent chow diet (CRF-1; Oriental Yeast Co., Ltd., Tokyo, Japan) was used to house Wistar rats (male, eight weeks old, 240-260 g; n = 58) under controlled conditions, similar to that in our previous study [13,29-31]. Effect sizes were calculated by power analysis (α = 0.05, power = 0.80) using G*Power 3 (Heinrich Heine University Düsseldorf, Düsseldorf, Germany), based on pilot von Frey test data, to determine a sufficient sample size. The analysis indicated that five rats per group were required.

The rats were euthanized at the times described below. We performed cervical dislocation after complete loss of consciousness induced by carbon dioxide inhalation, in accordance with the guidelines for the proper conduct of animal experiments established by the Science Council of Japan.

PRP gel preparation

Instead of using autograft blood, allograft blood was used in this investigation. Male rats, aged nine weeks, weighing approximately 400 g (n = 5), were anesthetized through intraperitoneal injection of a combination of 10 mg/kg xylazine hydrochloride and 100 mg/kg ketamine hydrochloride. Following anesthesia, 10 mL fresh blood was drawn transcardially with a syringe containing 1 mL acid-citrate-dextrose solution A (Terumo, Tokyo, Japan) to prevent clotting. The drawn blood was centrifuged (KN70; Kubota, Tokyo, Japan) at 300 g for 10 minutes to separate the plasma fraction from the erythrocytes, which was further centrifuged at 1000 g for 15 minutes to pellet platelets, as described previously [32,33]. Ultimately, 1 mL of PRP was obtained from 10 mL of peripheral blood. Subsequently, fresh PRP and whole blood platelet counts were determined with a hematology analyzer.

**Figure 1:**
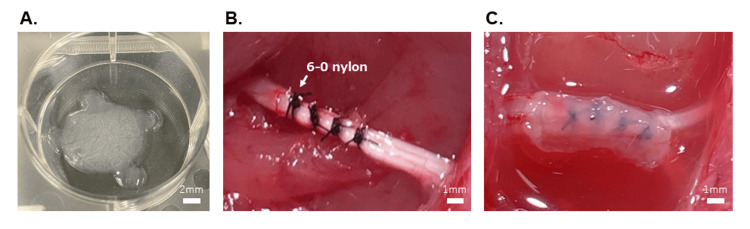
Preparation and grafting of PRP gel. (a) For the PRP-G group, 200 µL PRP was activated with CaCl2 in a 12-well plate, which resulted in the formation of the PRP gel. (b) Four ligatures, with 1-mm intervals between them, were loosely fastened to the sciatic nerve to produce the CCI sciatic nerve model. (c) The PRP gel wrapping process involves wrapping a PRP gel around the nerve, similar to vein wrapping. PRP: platelet-rich plasma; CCI: chronic constriction injury; PRP-G: PRP gel wrapping.

For activation of PRP, 200 μL PRP was mixed with 1 mEq/mL calcium chloride solution in a 12-well plate (Otsuka Pharmaceutical Co., Ltd., Tokyo, Japan) for 30 minutes, which was one-tenth of the total amount of PRP obtained. This procedure led to the formation of gel from the liquid form of PRP (Figure 1A). The gel was administered to the rats within one hour of preparation by the protocol described below. The prepared PRP samples were evenly distributed among the experiments.

Sciatic nerve injury rat model

We used a previously published technique to produce sciatic nerve CCI [34]. After anesthetizing the animals as described in the previous section, the biceps femoris and superficial gluteus maximus muscles were separated, and the right sciatic nerve was identified. We induced CCI by lightly fastening four ligatures around the sciatic nerve, spaced 1 mm apart, and tightening them until the corresponding hind limb exhibited a short twitch (Figure 1B). Rats (n = 28) were randomly assigned to two groups: PRP-G and control. The rats in the control group were subjected to CCI induction surgery only, whereas those in the PRP-G group underwent a PRP gel wrapping procedure after the CCI surgery, with freshly prepared PRP gel wrapped immediately around the sciatic nerve (Figure 1C).

Von Frey test

In accordance with our previous report [13,29-31], rats (n = 5 per group) underwent the von Frey test on day 0 (pre-surgery) and postoperative days (PODs) three, seven, and 14. Rats were randomly assigned by a third party, and the experimenter was blinded to group information. The von Frey test was carried out by applying a von Frey filament (Monofilament Kit; Smith & Nephew, Germantown, WI) to the hind paw at a 90° angle to the plantar surface after randomization and a one-hour acclimatization period in the test cage. The stimulus strength was slowly increased or lowered to find the withdrawal threshold. Baseline thresholds were determined three days before surgery. Based on a previous report [35], the Dixon nonparametric test was used to analyze the data [36].

Tumor necrosis factor-α and interleukin-6 gene (Tnf-α and Il-6) expression analysis

A method similar to that reported in our previous study was used to investigate the mRNA expression of *Tnf-α* and *Il-6* genes in the sciatic nerve [13,29-31]. The relative gene expression levels after sciatic nerve injury were measured with a separate group of rats (n = 5) known as the "normal group" that did not undergo CCI surgery or treatment before sciatic nerve resection. The rats (n = 5 per group, per timepoint) were anesthetized before the right sciatic nerve was resected (normal group); three, seven, and 14 days after wrapping; and after removal of scar tissue and ligatures from the resected nerves.

We used the polymerase chain reaction (PCR) primers (Hokkaido System Science Co., Ltd., Sapporo, Japan) shown in Table 1 to perform quantitative reverse transcription PCR (qRT-PCR) after RNA extraction and complementary DNA (cDNA) synthesis. A total volume of 25 μL was used for the qRT-PCR, which included 2 μL cDNA, sense and antisense primers (final concentration 0.2 mM), and 12.5 μL RNase solution (TB Green Premix Ex Taq; Takara, Kyoto, Japan). We performed qRT-PCR in a CFX-96 real-time PCR detection system (Bio-Rad, Hercules, CA) using the following parameters: initial denaturation at 95°C for one minute, 40 cycles of 95°C for five seconds, and 30 cycles of 60°C for 30 seconds. The expression of *Tnf-α* and *Il-6* mRNA was standardized to the levels of glyceraldehyde dehydrogenase (GAPDH). The relative expression (CCI or PRP-G/normal) was then calculated and compared between the two groups.

**Table 1:** Sequences of primers used for qRT-PCR in this study. Tnf-α: tumor necrosis factor alpha; Il-6: interleukin 6; GAPDH: glyceraldehyde dehydrogenase; bp: base pair; qRT-PCR: quantitative reverse transcription polymerase chain reaction.

Gene	Direction	Primer sequence (5¢–3¢)	Product size (bp)
Tnf-α	Sense	CTCTTCTCATTCCCGCTCGT	104
	Antisense	GGGAGCCCATTTGGGAACTT	
Il-6	Sense	CCAGTTGCCTTCTTGGGACT	167
	Antisense	TCTGACAGTGCATCATCGCT	
GAPDH	Sense	TGCCACTCAGAAGACTGTGG	129
	Antisense	TTCAGCTCTGGATGACCTT	

Histological evaluation of sciatic nerves

Tissue samples were obtained from rats (n = 4 per group) on POD seven, fixed in 5% glutaraldehyde and 4% paraformaldehyde (Karnovsky fixing solution). POD seven was selected as the most significant difference in pain behavior assessment. Each sciatic nerve was rinsed and re-fixed with 1% osmium tetroxide (OsO4) for three days, dehydrated with a 70-100% gradient of ethanol using a dehydration permeabilizer (Tissue-Tek V.I.P 6 AI; Sakura Finetek Japan Co., Ltd., Tokyo, Japan) and xylene; subsequently, paraffin blocks were prepared. Approximately 4 μm-thick sections prepared with a sliding microtome (LS113; Yamato Kohki Industrial Co., Ltd., Asaka, Japan) were stained with 1% toluidine blue (TB).

TB-stained specimens were photographed using a microscope (BX-43; Olympus Corporation, Tokyo, Japan) and a DP-22 (Olympus Corporation) imaging system at a magnification of 400×, and tiling images were combined using the multiple image alignment functions of CellSens (Olympus Corporation). The three measurements, density of myelinated nerve fibers, fiber diameter, and myelin sheath thickness, were performed with the image analysis software WinROOF version 7.2 (Mitani-shoji, Fukui, Japan) and a tablet input device (LCD Tablet PL-550; WACOM, Kasu, Japan).

Statistical analyses

All analyses were performed with GraphPad Prism 9 (GraphPad Software, San Diego, CA). Normality and variance of the data were evaluated using the Kolmogorov-Smirnov and F tests, respectively, to confirm that the data were normally distributed and that the variance between the groups was comparable. The statistical significance between the two groups was evaluated using a two-way ANOVA test followed by a Bonferroni post hoc test for the von Frey test and a Student's t-test for the other two group comparisons. P < 0.05 was considered statistically significant.

## Results

Confirmation of PRP

Blood cell measurement results of the whole blood and PRP are shown in Table 2. The mean platelet count in the peripheral whole blood was 927 ± 53.7 × 103/μL, and that in the PRP was 4158 ± 626 × 103/μL. On average, the platelet count in the PRP was approximately 4.4 times higher than that in the peripheral whole blood. In the PRP, white blood cells were 1200 ± 1157/μL, and hemoglobin was 0.1 ± 0.05 g/dL, both lower than those in peripheral whole blood. Hence, the PRP used in this study was leukocyte-poor PRP.

**Table 2:** Blood cell measurement results of whole blood and PRP. Data show mean ± standard deviation. PRP: platelet-rich plasma; WBC: white blood cells; Hb: hemoglobin; PLT: platelet.

	WBC (/µL)	Hb (g/dL)	PLT (10^3^/µL)
Whole blood	3940 ± 343	12.7 ± 0.6	927 ± 53.7
PRP	1200 ± 1157	0.1 ± 0.05	4158 ± 626
Concentration ratio			4.4 ± 0.58

Von Frey test

**Figure 2:**
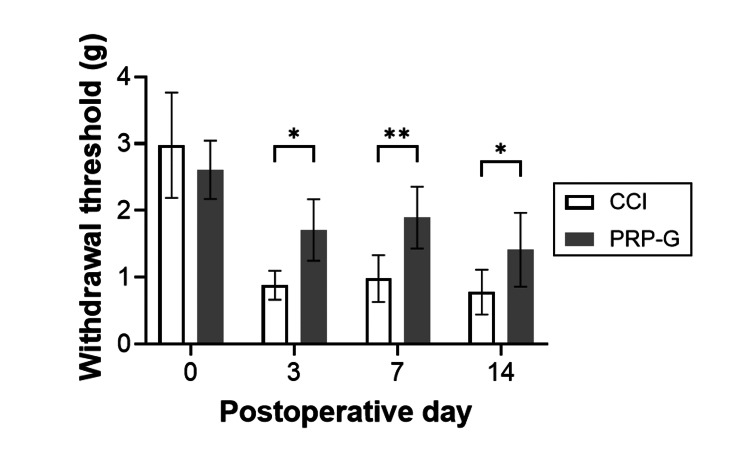
Withdrawal threshold in the CCI and PRP-G groups. Neuropathic pain was continually observed in the control group in the first two weeks following surgery. PRP gel wrapping after CCI causes a significant reduction in neuropathic pain compared with that in the CCI groups on days three, seven, and 14 following surgery (* P < 0.05, ** P < 0.01). Day 0 indicates before surgery. Data show mean ± standard error (n = 5, each group, each time point). Statistical analysis was performed using two-way ANOVA followed by a Bonferroni post hoc test. PRP: platelet-rich plasma; CCI: chronic constriction injury; PRP-G: PRP gel wrapping.

Neuropathic pain was observed in rats in both the CCI and PRP-G groups from POD one. In the CCI group, it persisted for the first two weeks post surgery. The withdrawal threshold was significantly higher in the PRP-G group from POD three, POD seven, and POD 14 (p-value = 0.048, 0.004, and 0.031, respectively) (Figure 2).

Tnf-α and Il-6 mRNA expression analysis

**Figure 3:**
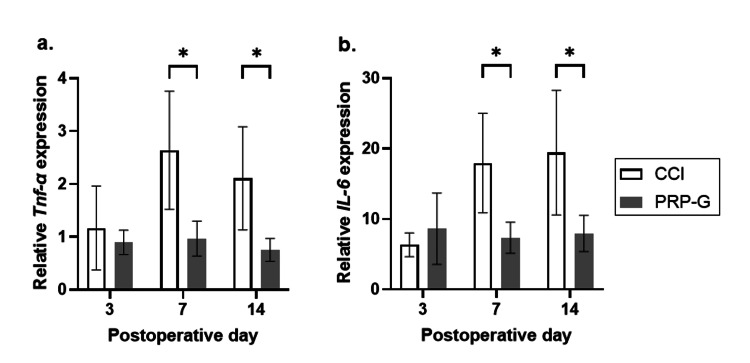
PRP gel wrapping on Tnf-α and Il-6 mRNA levels in the sciatic nerve after CCI. Effect of PRP gel wrapping on the expression of *Tnf-α* (a) and *Il-6* (b) in the sciatic nerve. PRP gel wrapping after CCI significantly reduced *Tnf-α* and *Il-6* mRNA levels compared with those in the CCI group on PODs seven and 14. * P < 0.05. Data show mean ± standard error (n = 5, each group, each time point). Statistical analysis was performed using multiple Student’s t-tests. PRP: platelet-rich plasma; CCI: chronic constriction injury; PRP-G: PRP gel wrapping; POD: postoperative day.

*Tnf-α* and *Il-6* mRNA expression was significantly lower in the sciatic nerves of the PRP-G group than in the CCI group on POD seven and 14 (*Tnf-α*: p = 0.037, 0.037; *Il-6*: p = 0.037, 0.047) (Figure 3).

Histological evaluation of sciatic nerves

**Figure 4:**
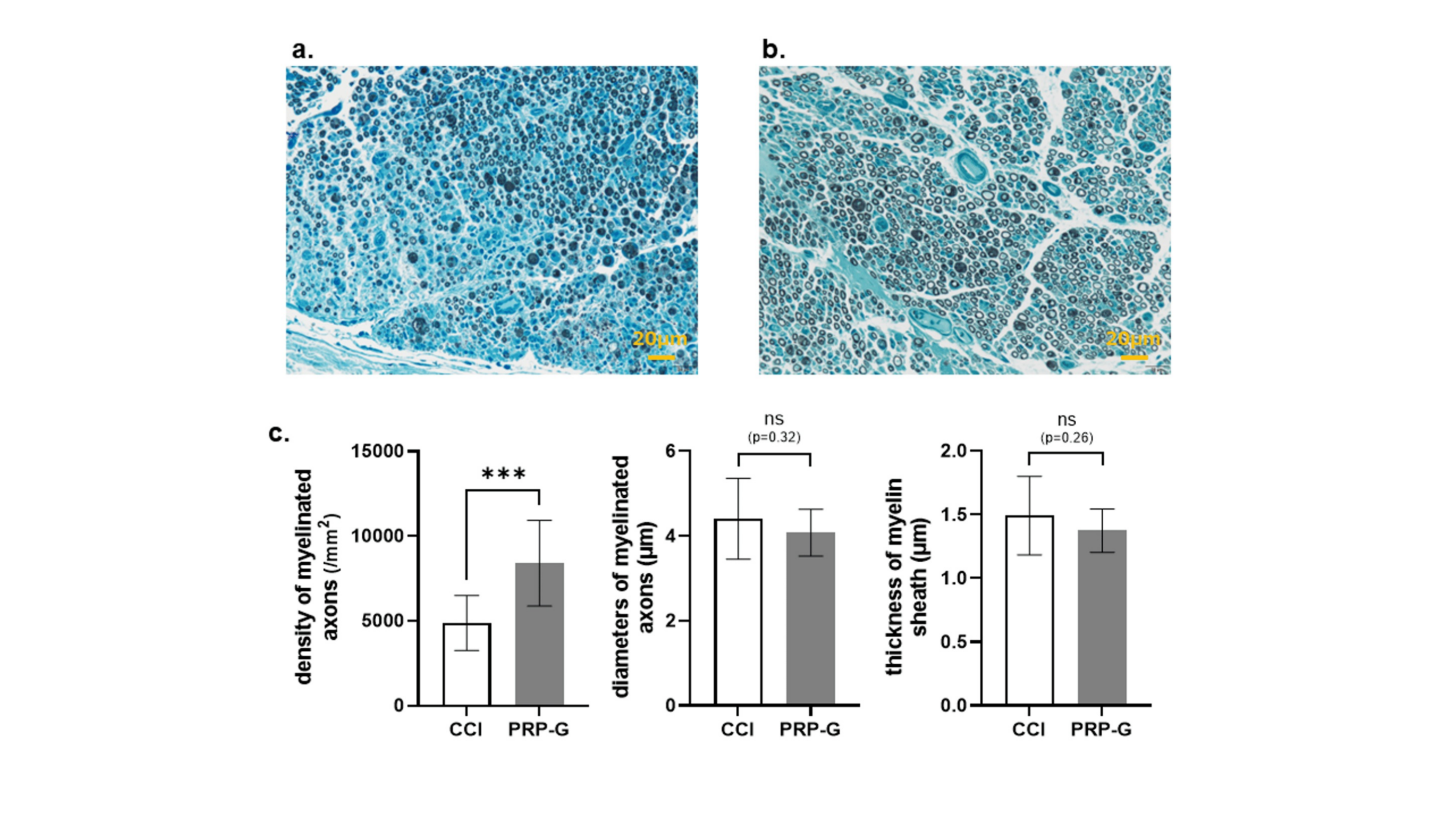
Histological evaluation of sciatic nerves of POD seven stained with toluidine blue. Micrographs (400×) of the CCI (a) and PRP-G (b) groups. Histomorphometry analysis was performed by calculating the mean myelinated nerve fiber density, the mean diameter of the myelinated nerve fiber, and the mean thickness of the myelin sheath between groups (c). * P < 0.05, ** P < 0.01, *** P < 0.001. Data show mean ± standard error (n = 5, each group). Statistical analysis was performed using Student’s t-test. Scale bar: 20 µm. PRP: platelet-rich plasma; CCI: chronic constriction injury; PRP-G: PRP gel wrapping; POD: postoperative day; ns: not significant.

Histological evaluation of sciatic nerves of POD seven stained with TB (400×) in the CCI group (Figure 4A) and PRP-G group (Figure 4B) is shown below. Density of myelinated axons was significantly higher in the PRP-G group than in the CCI group, but no significant differences were observed for diameters of myelinated axons and thickness of myelin sheath (p = 0.0009, 0.319, and 0.257, respectively) (Figure 4C).

## Discussion

In this study, the platelet enrichment of the PRP used was approximately 4.4 times higher than that of peripheral whole blood. The optimal PRP concentration for peripheral neuropathy or other orthopedic lesions is about four times that of whole blood to promote Schwann cell activity and nerve repair [37,38]. Therefore, the level of PRP enrichment used in this study was appropriate. The PRP gel was relatively elastic, making it easier to wrap it around the nerve closely.

Pain behavioral assessment showed significantly higher pain thresholds in the PRP-G group compared to the CCI group on PODs three, seven, and 14. In our previous study, bFGF-impregnated collagen sheets applied to rat CCI models were effective only until POD five [13]. In the present study, the effect of PRP gel lasted up to POD 14, suggesting that PRP gel may have a longer-lasting effect. The difference may be related to the action of bFGF alone versus the combined effect of multiple cytokines and growth factors present in PRP.

Several studies, including pathological validation, have demonstrated that PRP promotes regeneration of injured peripheral nerves. Most studies have used PRP-filled conduits for neural deficit models [27,28]. Using a rat sciatic nerve crush injury model, Zhu et al. [37] reported that PRP injection into the perineurium, at an appropriate concentration, promotes recovery of axonal function.

Although there is still room for debate, numerous reports show that PRP is effective in relieving nerve pain of peripheral origin. In clinical practice, it has been reviewed to be effective for treating pain from carpal tunnel syndrome [24], and its analgesic effect has been reported to last longer than that of steroids [23]. It has also been reported to be effective for diabetic neuropathic pain [39]. However, there have been few studies, such as ours, of animal experiments employing PRP or PRP gel in neuropathic pain-induced models. Behroozi et al. [40] reported that intra-spinally administered PRP suppressed neuropathic pain in a rat spinal cord injury model, indicating its role in the central nervous system. Although in a different context, Huang et al. [41] showed that injection of PRP into the scar area had analgesic effects in a rat model of burn-induced neuropathic pain.

To investigate the mechanism of the suppression of neuropathic pain at the molecular level by PRP gel wrapping, qRT-PCR was performed. On PODs seven and 14, the *Tnf-α* and *Il-6* mRNA expression in sciatic nerves was significantly lower in the PRP-G group than in the CCI group. PRP or PRP gel has not been shown to suppress neuronal expression of TNF-α and IL-6 in peripheral neuropathy animal models. In a study related to orthopedics, Sundman et al. found that co-culturing synovial membrane and cartilage with PRP significantly reduced TNF-α levels [42]. In a similar study, Moussa et al. [43] reported that PRP treatment of chondrocytes isolated from human osteoarthritic cartilage suppressed intracellular *Il-6* mRNA expression in chondrocytes.

Various pro-inflammatory cytokines produced by specific cells are essential for the development of persistent inflammation and pain after nerve damage. TNF-α, an essential mediator of chronic inflammation, significantly influences peripheral nerve damage and neuropathic pain.

Studies on CCI models have shown an association between increased levels of *Tnf-α* mRNA or protein in injured nerve and pain [44]. In our previous report, CCI caused a gradual increase in *Tnf-α* expression in the sciatic nerve from POD three to seven. However, this increase in POD seven was suppressed in the group that underwent decompression surgery on POD three compared to the group that did not undergo decompression. Immunostaining of the rat sciatic nerve isolated on POD seven showed that CD3 and TNF-α co-positive cells were significantly higher in the group that did not undergo decompression [29]. Although no decompression surgery was performed in this study, the CCI model is the same, and we believe that the significant reduction of *Tnf-α* gene expression in the nerve as a result of PRP treatment suppressed T cell infiltration [29].

T cells are a key regulator of the inflammatory response, and recent studies have revealed their involvement in the pathophysiology of peripheral nerve damage and chronic pain [45-49]. Damage to peripheral nerves enhances neuroinflammation and T-cell infiltration [49]. Additionally, in a CCI model, T cell insufficiency was shown to reduce thermal hyperalgesia [48].

In the present study, histology showed higher myelinated axon density in the PRP-G group than in the CCI group, with no difference in axon diameter or myelin thickness. This histological neuroprotective effect is not discussed in the literature because, as mentioned above, there are few studies similar to the present study. We postulate that it may be due to the inhibition of T cell infiltration into the sciatic nerve as a result of PRP wrapping. There are reports on PRP-induced nerve regeneration demonstrating that PRP promotes axon elongation via Schwann cells [19,27]. Thus, Schwann cells may also play a neuroprotective role in the present model.

Taken together, the inhibitory effect of PRP gel on neuropathic pain may involve the suppression of TNF-α and IL-6 expression in the nerves. The results of this study demonstrate the clinical use of PRP for the treatment of peripheral nerve damage.

This study has some limitations. First, we could not generate gels without the PRP component; hence, the outcomes of PRP gel observed in this study may also include some effects of the wrapping process itself. However, in our previous study with a comparable CCI model that induced potent neuropathic pain, we had wrapped the nerve with collagen sheets soaked in saline, but there was no improvement in pain [13]. Experiments using substances that can convert both saline solution and PRP to gel and are suitable for human use are necessary in the future. Second, this study did not perform *Tnf-α* and *Il-6* immunohistochemistry or measure T cell-related markers. The molecular mechanism by which PRP gel suppresses the expression of *Tnf-α* and *Il-6* genes in nerves and maintains its effect for a relatively long period remains unclear, and further research is needed. Third, electrophysiological testing has not been performed, and evaluation will be necessary in the future. Fourth, in this study, we did not measure the growth factor concentration of PRP because it was prepared using the same method as in our previous report, and the primary growth factor contents were reported [33]. Fifth, this study used leukocyte-poor PRP, but further investigation using leukocyte-rich PRP is necessary. Sixth, to standardize conditions, we used blood from the same species, which may have influenced inflammatory responses. Seventh, this study was conducted using rodents, and additional research is required before clinical application in humans can be considered.

## Conclusions

In this study, PRP gel wrapping applied to injured nerves in a rat CCI model was found to suppress the expression of the *Tnf-α* and *Il-6* genes in the sciatic nerve, suggesting the potential for long-term pain relief. PRP gel wrapping does not require a carrier and may have potential as an adjunctive treatment for peripheral nerve injury in the future; however, this study was conducted using rodents, and further investigation is necessary.
